# Ultrasonic Elastography Research Based on a Multicenter Study: Adding Strain Ratio after 5-Point Scoring Evaluation or Not

**DOI:** 10.1371/journal.pone.0148330

**Published:** 2016-02-10

**Authors:** Wen-Jie Mu, Wen-Jing Zhong, Ji-Yi Yao, Lu-Jing Li, Yu-lan Peng, Yi Wang, Li-sha Liu, Ying Xiao, Shou-jun Liu, Chang-jun Wu, Yu-xin Jiang, Shyam Sundar Parajuly, Ping Xu, Yi Hao, Jing Li, Bao-Ming Luo, Hui Zhi

**Affiliations:** 1 Department of Ultrasound, Sun Yat-sen Memorial Hospital, Sun Yat-sen University, Guangzhou, Guangdong Province, China; 2 Department of Diagnostic Ultrasound and The National Key Discipline of Medical Imaging and Nuclear Medicine, West China Hospital of Sichuan University, Chengdu, Sichuan Province, China; 3 Department of Ultrasound, Huashan Hospital, Fudan University, Shanghai, Zhejiang Province, China; 4 Department of Ultrasound, Tumor Hospital Affiliated to Xinjiang Medical University, Urumqi, Xinjiang Province, China; 5 Department of Ultrasound, Xiangya Hospital, Central-South University, Changsha, Hunan Province, China; 6 Department of Ultrasound, ShengJing hospital of China Medical University, Shenyang, Liaoning Province, China; 7 Department of Ultrasound, The First Affiliated Hospital of Harbin Medical University, Harbin, Heilongjiang Province, China; 8 Department of Ultrasound, Peking Union Medical College Hospital, Chinese Academy of Medical Sciences and Peking Union Medical College, Beijing, Hebei Province, China; Semmelweis University, HUNGARY

## Abstract

**Background:**

This study aimed to confirm whether strain ratio should be added after evaluation of lesions with 5-point elasticity scoring for differentiating benign and malignant breast lesions on ultrasonographic elastography(UE).

**Materials and Methods:**

From June 2010 to March 2012, 1080 consecutive female patients with breast lesions were recruited into a multicenter retrospective study, which involved 8 centers across China. Each institutional ethic review board approved the study, and all the patients gave written informed consent. All the patients underwent the UE procedure and the strain ratios were calculated and the final diagnosis was made by histological findings. The sensitivity, specificity, accuracy, PPV and NPV were calculated for each of the two evaluation systems and the areas under the ROC curve were compared.

**Results:**

The strain ratios of benign lesions (mean, 2.6±2.0) and malignant lesions (mean,7.9±5.8) were significantly different (p <0.01). When the cutoff point was 3.01, strain ratio method had 79.8% sensitivity, 82.8% specificity, and 81.3% accuracy, while the 5-point scoring method had 93.1% sensitivity, 73.0% specificity, and 76.8% accuracy. The areas under the ROC curve with the strain ratio method and 5-point scoring method were 0.863 and 0.865, respectively(p>0.05). The strain ratio method shows better a diagnosis performance of the lesions with elasticity score 3 and 4.

**Conclusions:**

Although the two UE methods have similar diagnostic performance, separate calculation of the strain ratios seems compulsory, especially for the large solid breast lesions and the lesions with elasticity score 3 and 4.

## Introduction

As the incidence of breast cancer rises continuously, early breast cancer detection becomes increasingly important[1]. For physical examine, palpation is a highly applied way for surgeons in the clinical routine, yet as a subjective method, the size and location of the lesion require rich experience of the practitioner. When it comes to imaging examination, ultrasonography become an important evaluation of the first time screening as a convenient and noninvasive diagnoses method. In earlier sonoelastographic studies, diagnoses were made according to the differences in the size, shape, boundary of lesions on B-mode sonography[2,3]. For the concept that harder masses are more likely to be malignant, in the last decade, different ultrasonographic elastography(UE) diagnosis systems have been developed to determine the relationship between different structures and their tissue inherent elasticity for diagnosing malignant tumors UE, which involves the visual display of tissue stiffness, is a newly developed dynamic technique widespread used for detecting pathological tissue alterations in vivo. Since the method of UE was developed in 1980s[4], it is becoming an efficient examination in the diagnosis of liver lesions[5], thyroid cancer[6] and breast cancer[4–7]. As usual, the 5-point scoring system is used as an evaluation system when we diagnose a breast lesion, which scales a lesion by different color map which reflected by the stiffness of the focal tissue. Different scholars have different standards to score a lesion, score 1–3 is benign and 4–5 is malignant[8]. In our practice of this study, the diagnostic criteria was the new standard of 5-point scoring suggested by zhi et al[9]. Unfortunately, in practice, we found that just as the palpation, the score judgment of a lesion might be influenced by multiple subjective factors, that means different doctors will not generate a consistent diagnosis of a same lesion under the same conditions. In this circumstance, Waki et al present a new diagnosis system using the strain ratio measurement[10]. By this way, stiffness of breast lesions could be semi-quantitative calculated with the same depth of breast tissue as the reference and the subjective bias might be controlled[11].

In this multiple center study generated by 8 centers across China, we first scored the breast lesions by a 5-point scoring method and then calculated the strain ratios of the lesions. Our purpose was to investigate whether the strain ratio evaluation should be added after evaluation of lesions with 5-point elasticity scoring when differentiating breast lesions.

## Materials and Methods

This retrospective study was designed as a multi-center trial that involved 8 centers from 4 geographic regions across China (north, south, central, and west). Each institutional ethic review board approved the study: *Multicenter Study of breast Ultrasonic Elastography*. And all the centers used the same forms, all the patients gave written informed consent. Each center started the study after 6 months of experience of using UE. (S1 File)

### Patients

The inclusion criteria of patients were the following: female patients of at least 18 years of age, with a solid lesion in the breast through examining with conventional ultrasound and UE, and with histological confirmation in all cases. The data collection was from June 2010 to March 2012. 1080 breast lesions in 1080 consecutive patient (age range 18–88) were evaluated. All patients were examined with B-mode sonography and UE. Patients were excluded if the distance between the front boundary of the lesion and the skin was >2cm, because deeper lesions would have very little deformation.

### Methods

All images were acquired with a Hitachi EUB-900 (Hitachi Medical, Tokyo, Japan) or HI VISION PREIRUS (Hitachi Medical) including software with a combined auto-correlation and a Linear-array probe operating at 7.5–13 MHz, and both the conventional and the elastographic studies were performed by 2 radiologists with more than 5 years of experience in breast imaging and 3–48 months of experience with UE.

Firstly, On B-mode sonography, images were displayed in both transverse and longitudinal scans to estimate the lesion features together with sufficient surrounding tissue were taken in the region of interest. The BIRADS system[12] was used to obtain a summative evaluations of the B-mode sonography features of each lesion.

Then, on the real time UE, sonoelastographic images were obtained by means of color type as maps which translucently superimposed on the B-mode images in vivo. The region of interest mostly extended from the subcutaneous fat layer to the greater pectoral muscle to scan sufficient breast tissue. Elasticity images were obtained with appropriate compression when we pressed the probe repetitively of a slight power periodically. To obtain a optimal elasticity image, the process was repeated when the pressure indicator bar displayed an index of 3–4 until a stable image was acquired.

The UE images were illustrated in a color map, red, blue and green respectively indicated soft, intermediate and hard elasticity. In the 5-point scoring evaluation of the sonoelastographic images, the new standard lesion classification was proposed by Zhi et al[9], for this 5-point classification, a score of 1 indicated even strain for the entire lesion (ie, the entire lesion was evenly shaded in green). A score of 2 indicated strain in most of the lesion, with a little areas of no strain (ie, the lesion was shaded green in major with a little blue). A score of 3 indicated strain and no strain almost half in half of the lesion (ie, the lesion was green and blue half in half). A score of 4 indicated no strain or a little strain in the entire lesion (ie, the entire lesion was blue, or blue in major with a little green). A score of 5 indicated no strain or a little strain in the entire lesion and in the surrounding area (ie, the entire lesion and its surrounding area were blue, or blue in major with a little green) If a lesion’s score was between 1 and 3, it was categorized as benign. If a lesion was assigned score of 4 or 5, it was categorized as malignant.

To decide the score of each lesion, all the images were retrospectively reviewed by 2 radiologists, who had at least 3 years’ experience in breast UE. Both readers were blinded to patient identification, clinical history, other imaging results, and pathologic findings. First, the 2 radiologists analyzed the images respectively, then together they discussed the final results.

In the evaluation system of the strain ratio method, the stiffness of the lesions were estimated by strain ratio measurement with a same-depth area as an internal reference[10,11] For this purpose, the region of interest including the lesion was expressed as A, and the region of interest including the same-depth area was expressed as B, depending on the lesion stiffness compared with the surrounding normal area in each case, the strain ratio was automatically calculated as a B/A ratio.

The histology of all the lesions was established with an ultrasound-guided, 14-gauge, automated gun core biopsy within 48 hours of ultrasound examinations or excision biopsy. Only core biopsy results with a definitive diagnosis were accepted, those that were with undefined diagnosis underwent excision. The histology of the excised specimen was used for analysis. All samples obtained were sent for histologic study and were analyzed by specialized breast pathologists with at least 15 years of experience.

### Statistical Analysis

Differences among the scores and strain ratios for benign and malignant breast lesions were assessed with the Student *t* test. Receiver operating characteristic curves(ROC) were used to describe and compare the diagnostic performances of the 5-point scoring and strain ratio methods. To suggest optimal strain ratio for differentiation between benign and malignant masses, the best cutoff point obtained by calculating the Youden’s index, then the *z* test was used to compare the area under the curve (AUC). The accuracy, sensitivity, specificity and positive predictive values was compared by McNemar’s test.

Statistical analyses were performed with SPSS version 13 software (SSPS Inc, Chicago, IL) and MedCalc (MedCalc Software, Mariakerke, Belgium). Two-tailed p < .05 was considered statistically significant.

## Results

Of the 1080 breast lesions, 580(53.7%) were benign, and 500(46.3%) were malignant. The diameters of malignant lesions and benign lesions were 5~88(21.4±10.2)mm and 3.3~51.3(15.9±7.9)mm. Histological diagnosis were summarized in Table 1.

**Table 1:** Histological diagnosis in 1080 patients with benign or malignant breast lesions.

Benign lesions(n = 580)	n	Malignant(n = 500)	n
Fibroadenoma	356	Invasive ductal carcinoma	388
Fibroadenomatous hyperplasia	60	Ductal carcinoma in situ	55
Papilloma	52	Invasive lobular carcinoma	8
Adenopathy	34	Mucinous carcinoma	8
Phyllodes tumor	25	Invasive papillocarcinoma	7
Chronic inflammation	17	Neuroendocrine carcinoma	7
Atypical ductal hyperplasia	5	Intraductalpapillary carcinoma	6
Tubular adenoma	5	Invasive adenocarcinoma	5
Juvenilehyalincfibromatosis	4	Medullary carcinoma	3
Lipoma	2	Lymphoma	2
Other benign lesions	20	Other malignant lesions	11

### Strain ratio of breast lesions

**Fig 1:**
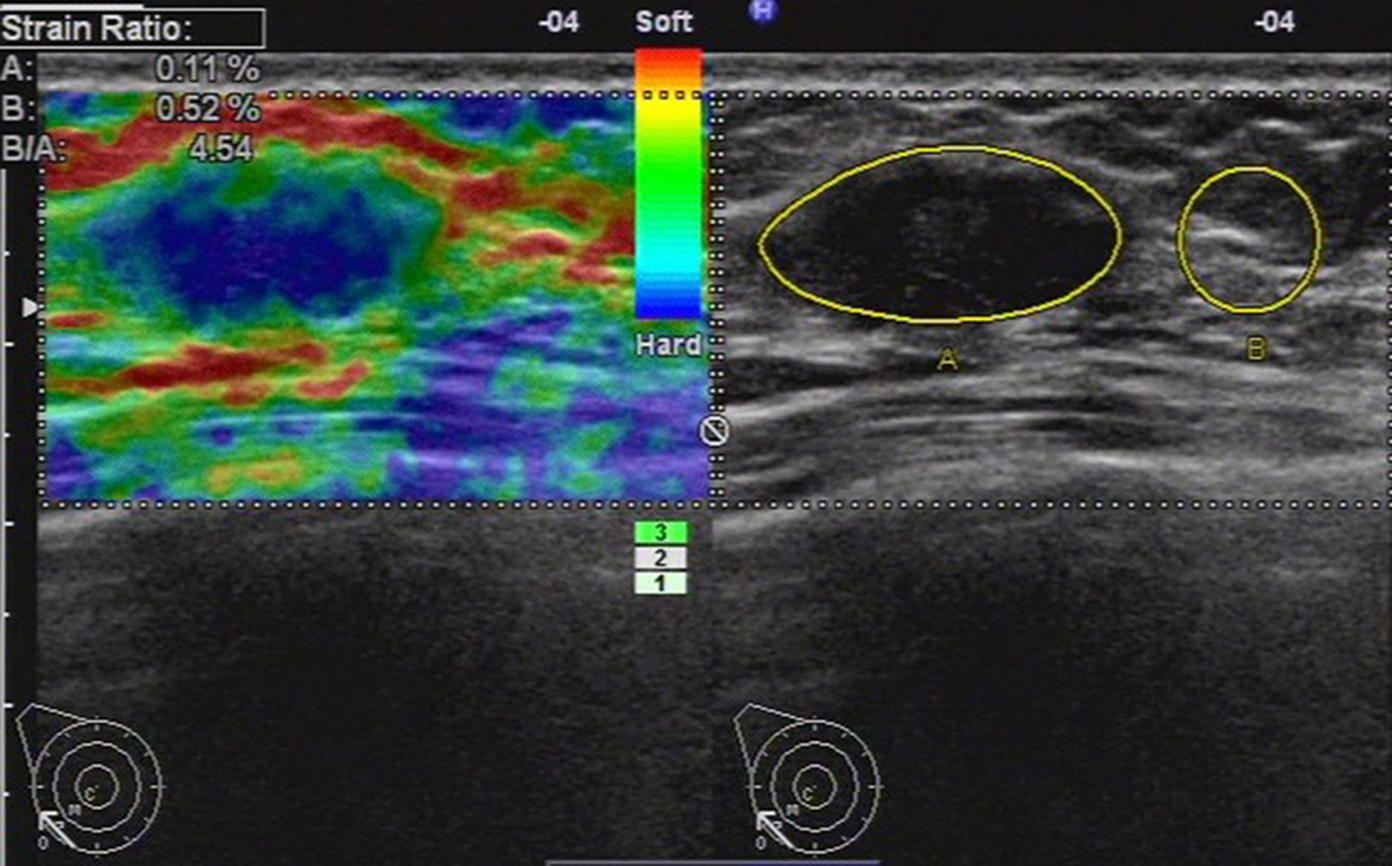
Ultrasonic elastographic image of a papillocarcinoma in a 46-year-old woman. The lesion was scored 4 with the 5-point scoring method. The strain ratio was 4.54 with the strain ratio method at the same breast tissue depth as the reference.

**Fig 2:**
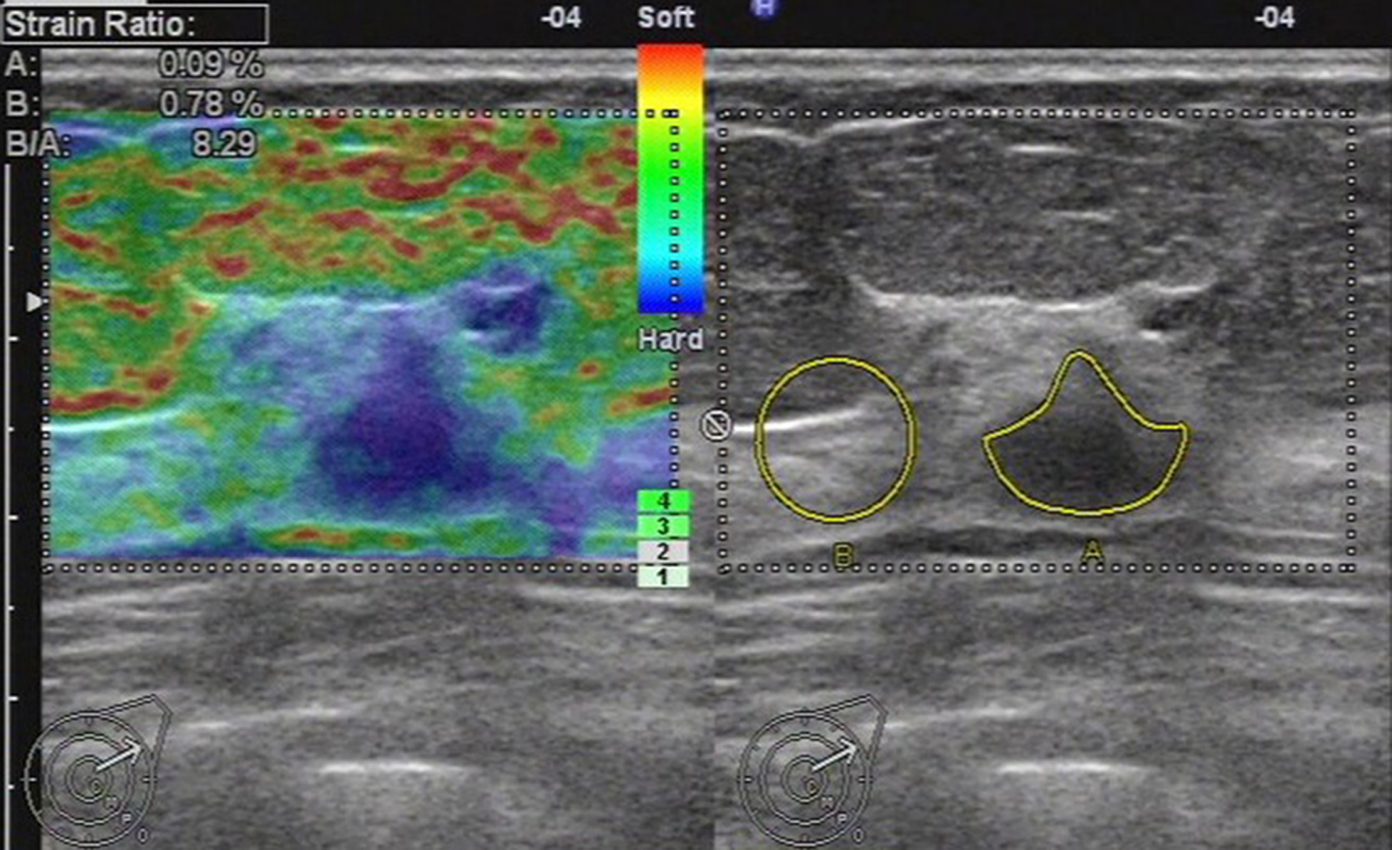
Ultrasonic elastographic image of an invasive lobular carcinoma in a 77-year-old woman. The lesion was scored 4 with the 5-point scoring method. The strain ratio was 8.29 with the strain ratio method at the same breast tissue depth as the reference.

For all 1080 lesions which were assessed the strain ratio by longitudinal direction, the mean strain ratios of benign and malignant lesions were 2.6±2.0, 7.9±5.8, respectively. In addition, extra strain ratio were calculated by the transverse direction for 481 lesions (228 malignant/253 benign), the mean strain ratios of benign and malignant lesions were were 2.4±1.9 and 9.8±8.4, respectively. For both longitudinal direction and transverse direction, strain ratios for malignant lesions were higher than the corresponding values for benign ones (all p < .05). The differences between the strain ratio assessed by the two directions had no statistically significant differences(p = 0.554). For the 580 malignant lesions in our study, there were 411 invasive breast cancer and 84 non-invasive breast cancer proved by gold standard diagnsis. The mean strain ratio of the non-invasive breast cancer (Fig 1) was 5.9±6.9, which was lower than 8.7±6.9 of the invasive breast cancer (Fig 2), the differences between them were statistically significant (p = 0.0001).

The ROC curve was used to assess the diagnosis performance of the strain ratio method and the AUC was 0.865, the maximum Youden’s index was 0.624. As the best cutoff point is defined when it attains the maximum of value of Youden’s index, the best cutoff point in our study was 3.01. With this best cutoff point, the sensitivity, specificity, accuracy, PPV and NPV of the SR method were 79.6%, 82.8%, 81.3%, 79.9%, and 82.5%.

### Comparison of 5-Point scoring method and strain ratio method with UE

**Fig 3:**
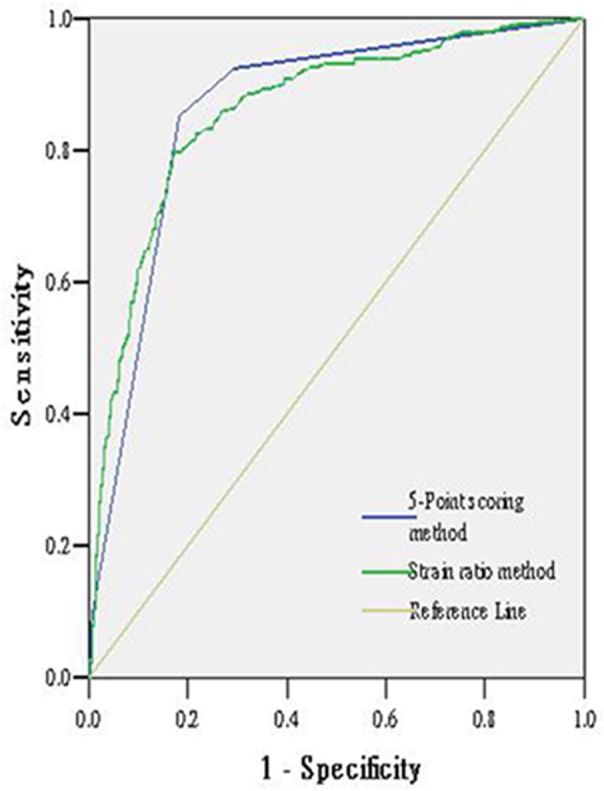
Receiver operating characteristic curves for the 5-point scoring method and the strain ratio method.

According to the ROC curves, the AUCs were 0.863 for 5-point scoring method and 0.865 for the strain ratio method (Fig 3). There were no significant differences between the two methods (p>0.05). Table 2 shows the comparison of sensitivity, specificity, accuracy, NPV and PPV of the two method. The specificity of the strain ratio method was 82.8% and that is 73% of the 5 point scoring method. There were significant differences between them (p = 0.020). Others had no significant differences (p>0.05).

**Table 2:** Comparison of sensitivity, specificity, accuracy, PPV and NPV obtained using strain ratio method and 5-point scoring method of the differentiation of benign and malignant breast lesions.

Lesions	Method	Sensitivity(%)	Specificity(%)	Accuracy(%)	PPV(%)	NPV(%)	AUC
Total lesions	SR	79.6	82.8*	81.3	79.9	82.5	0.863
	5-Score	93.1*	73.0	76.8	80.0	86.6	0.865
Small lesions	SR	75.9	80.9	80.0	47.8	93.6	0.917
	5-Score	93.1	73.0	76.8	44.3	97.9	0.885
Median lesions	SR	81.1	83.4	82.4	77.9	85.9	0.942
	5-Score	84.2	83.7	83.9	82.4	86.8	0.915
Large lesions	SR	85.0	93.8	84.5	90.1	77.3	0.918
	5-Score	78.9	82.4	80.2*	90.2	69.2	0.907*

NPV, negative predictive value; PPV, positive predictive value; AUC, area under the ROC curve.

*P < .05 verse another diagnostic system.

### Further Analysis of diagnosis performance of strain ratio method and 5-point scoring method in different size groups

**Fig 4:**
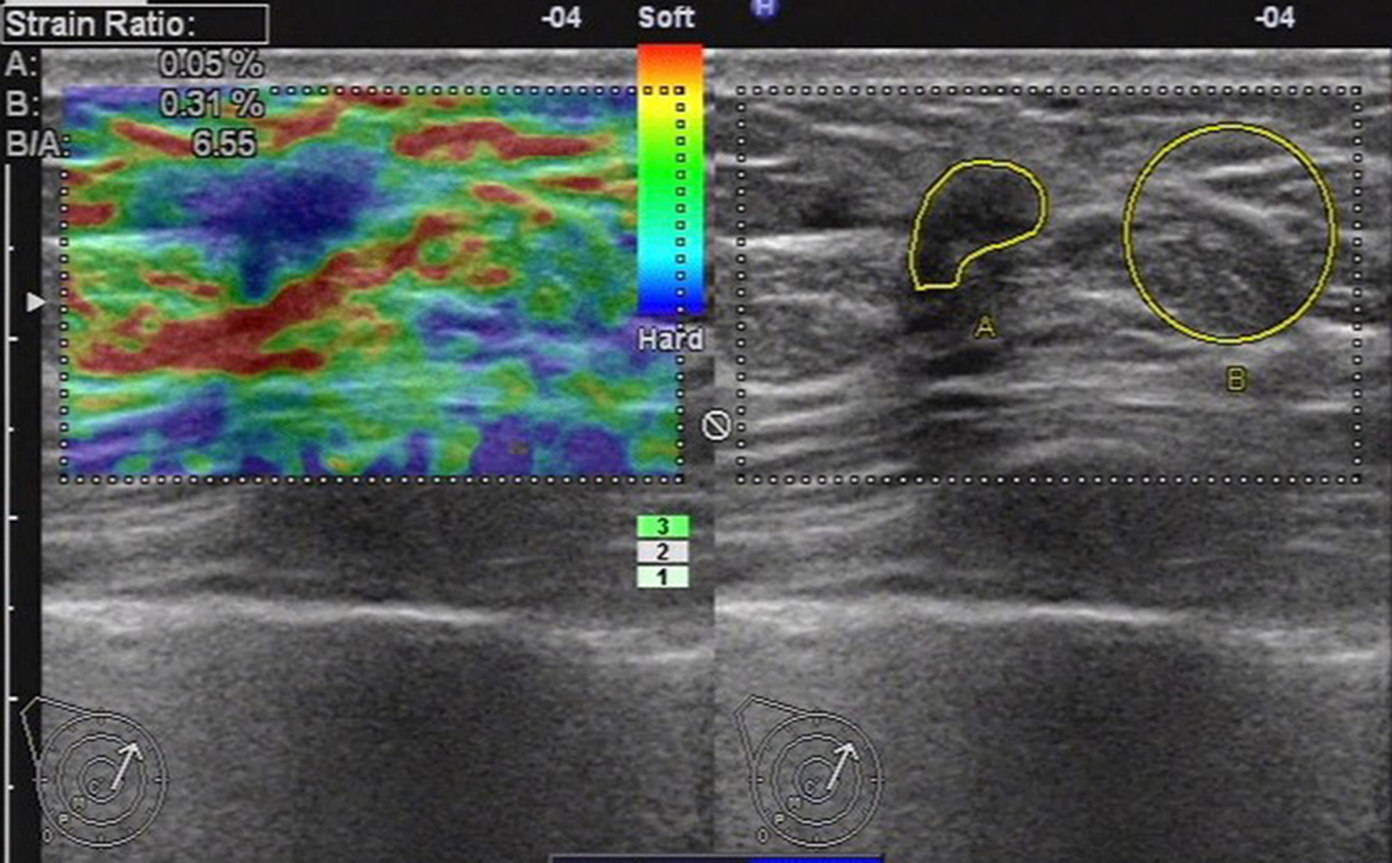
Ultrasonic elastographic image of an IDC in a 47-year-old woman. The lesion was scored 5 with the 5-point scoring method and the maximum diameter of the lesion was 8.2mm. The strain ratio was 6.55 with the strain ratio method at the same breast tissue depth as the reference.

For the 1080 lesions which were calculated the strain ratio in this poly-center study, of the lesion in the <10mm size group, 29 were malignant(18.7%), and 126 were benign(81.3%). The AUC obtained with the strain ratio method was 0.863, and that obtained with the 5-point scoring method was 0.865 (Fig 4). There were no significant differences between the diagnostic performance of the two methods (p = 0.683).

Of the lesion in the ≤10-20mm group, 222 were malignant(42.0%), and 307 were benign(58.0%). The AUC obtained with the strain ratio method was 0.917, and that obtained with the 5-point scoring method was 0.885. There were no significant differences between the diagnostic performance of the two methods (p = 0.470).

**Fig 5:**
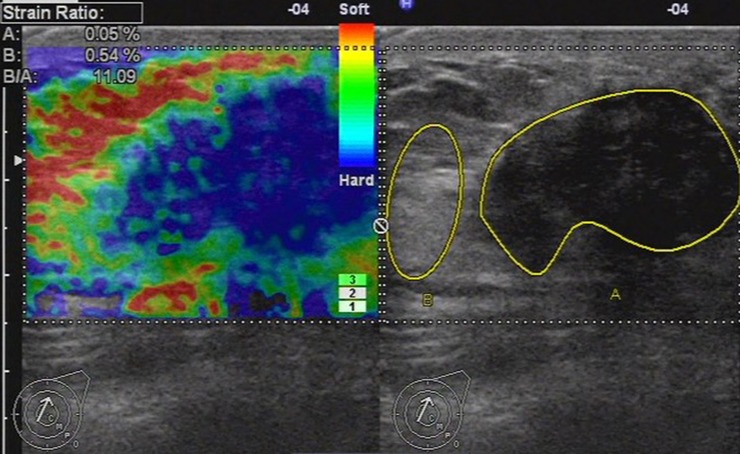
Ultrasonic elastographic image of an IDC in a 52-year-old woman. The lesion was scored 5 with the 5-point scoring method and the maximum diameter of the lesion was 28.1mm. The strain ratio was 11.09 with the strain ratio method at the same breast tissue depth as the reference.

Of the lesion in the ≥20mm group, 246 were malignant(66.8%), and 142 were benign(33.2%). The AUC obtained with the strain ratio method was 0.918, and that obtained with the 5-point scoring method was 0.907 (Fig 5). There were significant differences between the diagnostic performance of the two methods (p = 0.035).

Table 2 shows the sensitivity, specificity, accuracy, NPV and PPV of the strain ratio method and 5-point scoring method in each group, respectively. For the lesion in the ≥20mm group, the accuracy of the strain ratio method was 84.5% and that was 80.2% of the 5 scoring method. There were significant differences between them (p = 0.037). Other diagnostic index had no significant differences (p>0.05).

### Correlation between strain ratios and elasticity score

There was a good correlation between strain ratio method and elasticity score (Table 3). Lesions of elasticity score 1 and 2 had low overall mean strain ratios while those of elasticity score 4 and 5 had high overall mean strain ratios.

**Table 3:** Correlating the median strain ratios of lesions with each elasticity score.

Elasticity scores	Lesions		Median strain ratio
	Benign	Malignant	All
1	97	2	1.07 (0.35S.D.)
2	322	40	1.54 (0.65S.D.)
3	54	31	2.49 (1.49S.D.)
4	104	382	5.28 (8.75S.D.)
5	3	45	9.05 (10.50S.D.)

### Analysis of diagnosis performance to evaluate the strain ratios of lesions with each elasticity score

**Fig 6:**
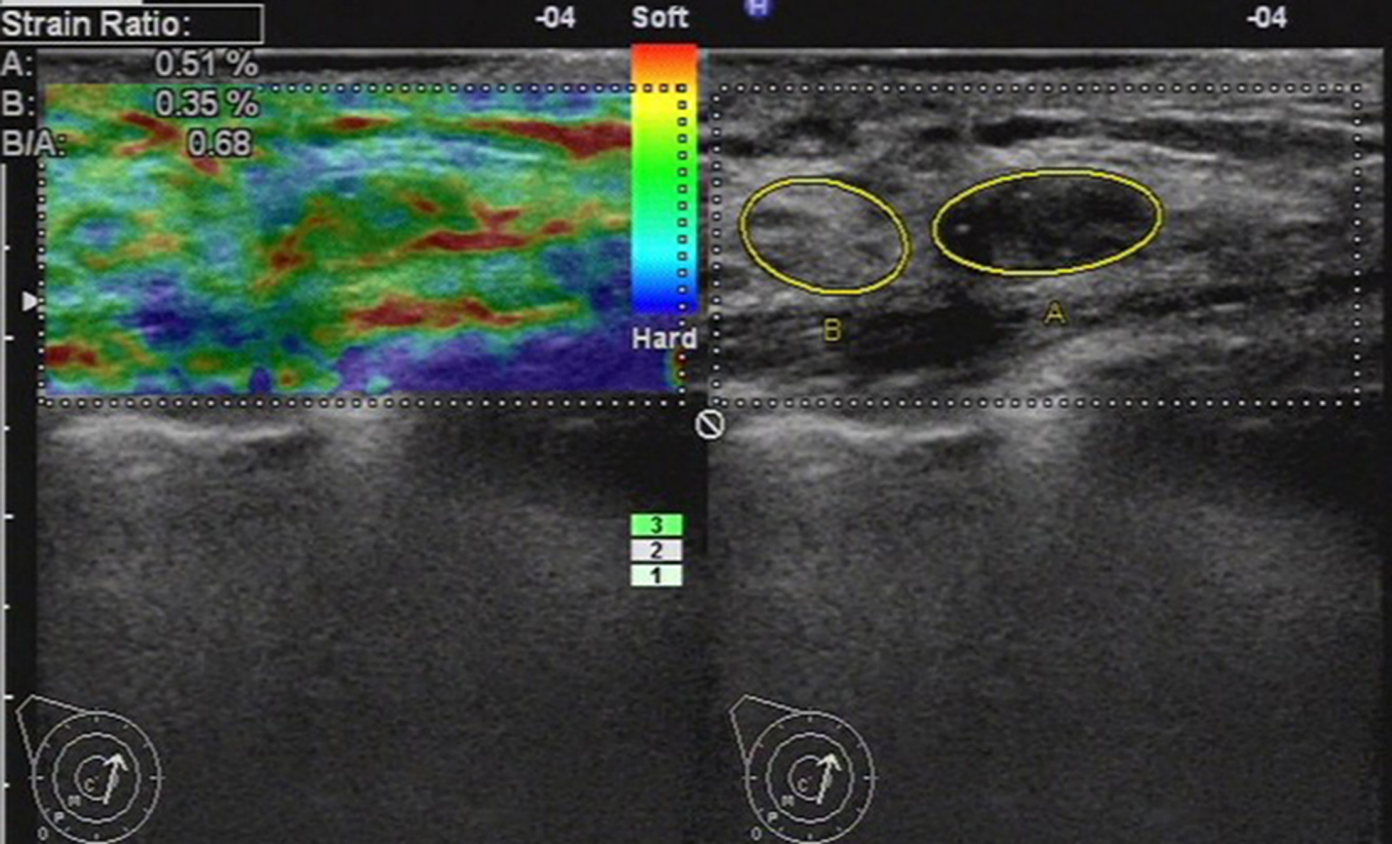
Ultrasonic elastographic image of a fibroadenomatous hyperplasia in a 39-year-old woman. The lesion was scored 1 with the 5-point scoring method. The strain ratio was 0.68 with the strain ratio method at the same breast tissue depth as the reference.

99 lesions were included in the elasticity score 1 group. According to histology, 98.0% (97 of 99) were benign, which were under strain ratio 3.01 totally. 2.0% (2 of 99) were malignant, which were exceeding strain ratio 3.01. Altogether the strain ratio method diagnosed 97 lesions (98.0%) correctly for score 1 group (Fig 6).

**Fig 7:**
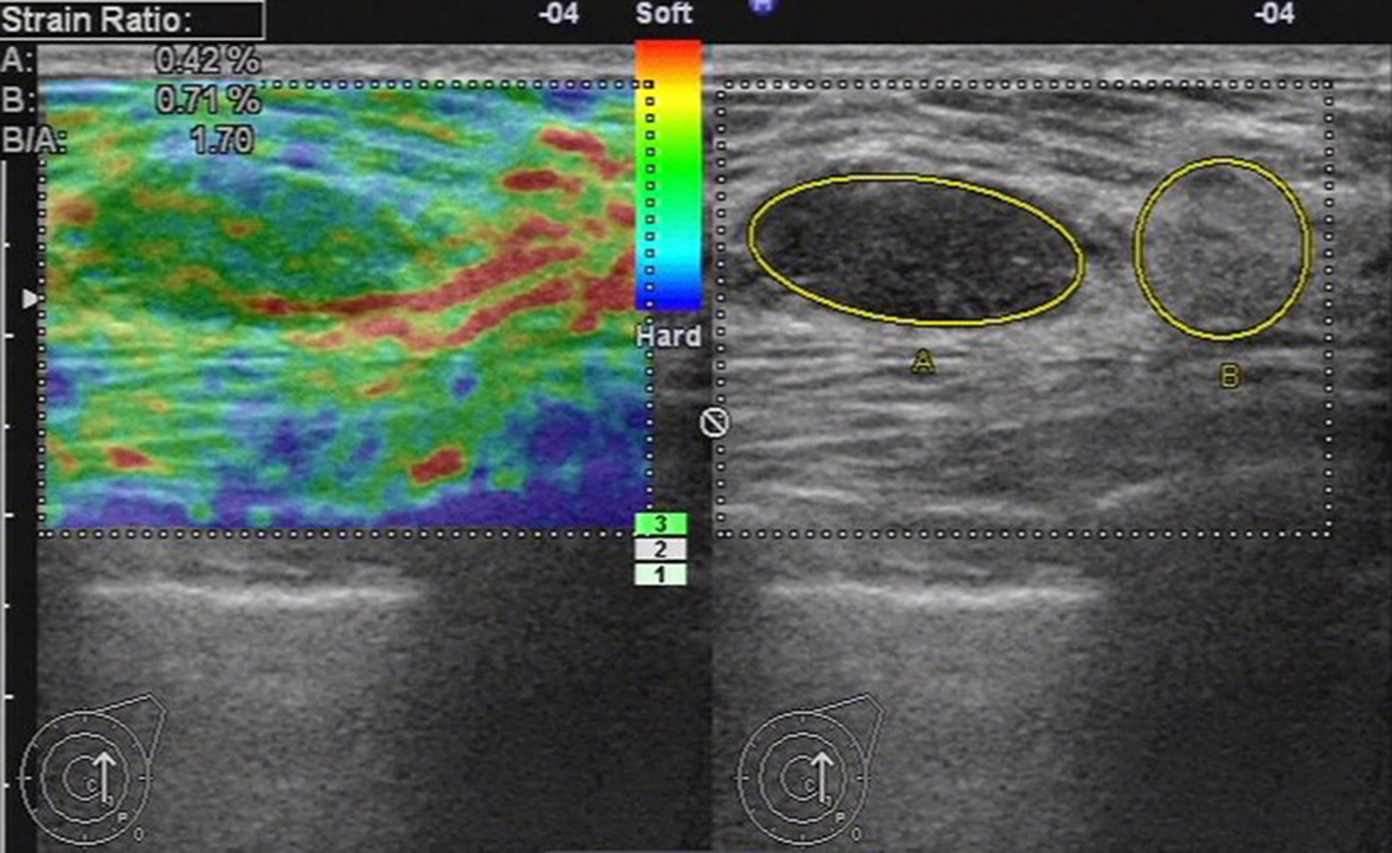
Ultrasonic elastographic image of a fibroadenoma in a 38-year-old woman. The lesion was scored 2 with the 5-point scoring method. The strain ratio was 1.70 with the strain ratio method at the same breast tissue depth as the reference.

362 lesions were included in the elasticity score 2 group. According to histology, 89.0% (322 of 362) were benign, among which 315 lesions were under strain ratio 3.01. 11.0% (40 of 362) were malignant, among which 10 lesions were exceeding strain ratio 3.01. Altogether the strain ratio method diagnosed 318 lesions (87.8%) correctly for score 2 group (Fig 7).

**Fig 8:**
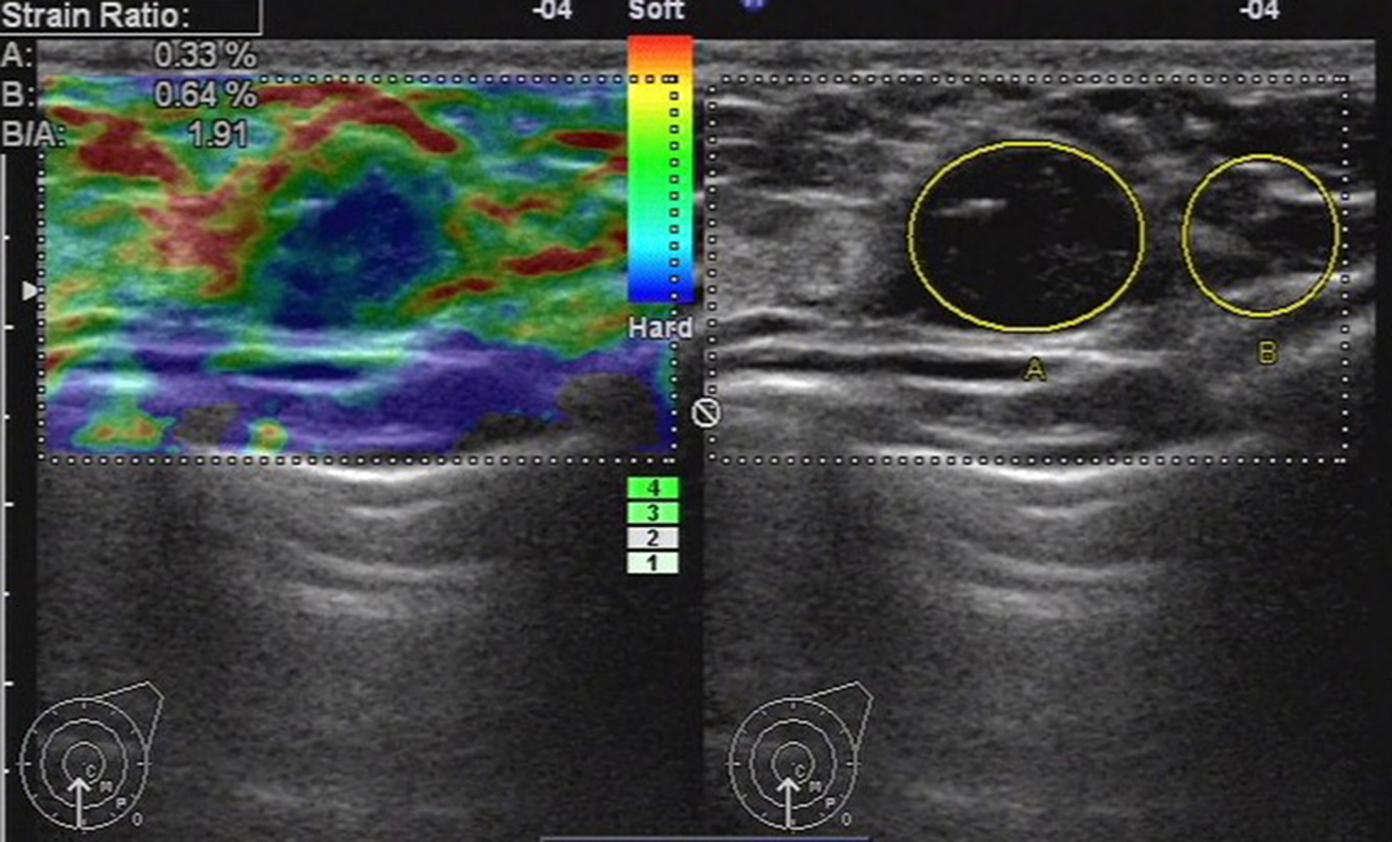
Ultrasonic elastographic image of a papiloma in a 34-year-old woman. The lesion was scored 3 with the 5-point scoring method. The strain ratio was 1.91 with the strain ratio method at the same breast tissue depth as the reference.

85 lesions were included in the elasticity score 3 group. According to histology, 63.5% (54 of 85) were benign, among which 40 lesions were under strain ratio 3.01. 36.5% (31 of 85) were malignant, among which 14 lesions were exceeding strain ratio 3.01. Altogether the strain ratio method diagnosed 54 lesions (63.5%) correctly for score 3 group (Fig 8).

**Fig 9:**
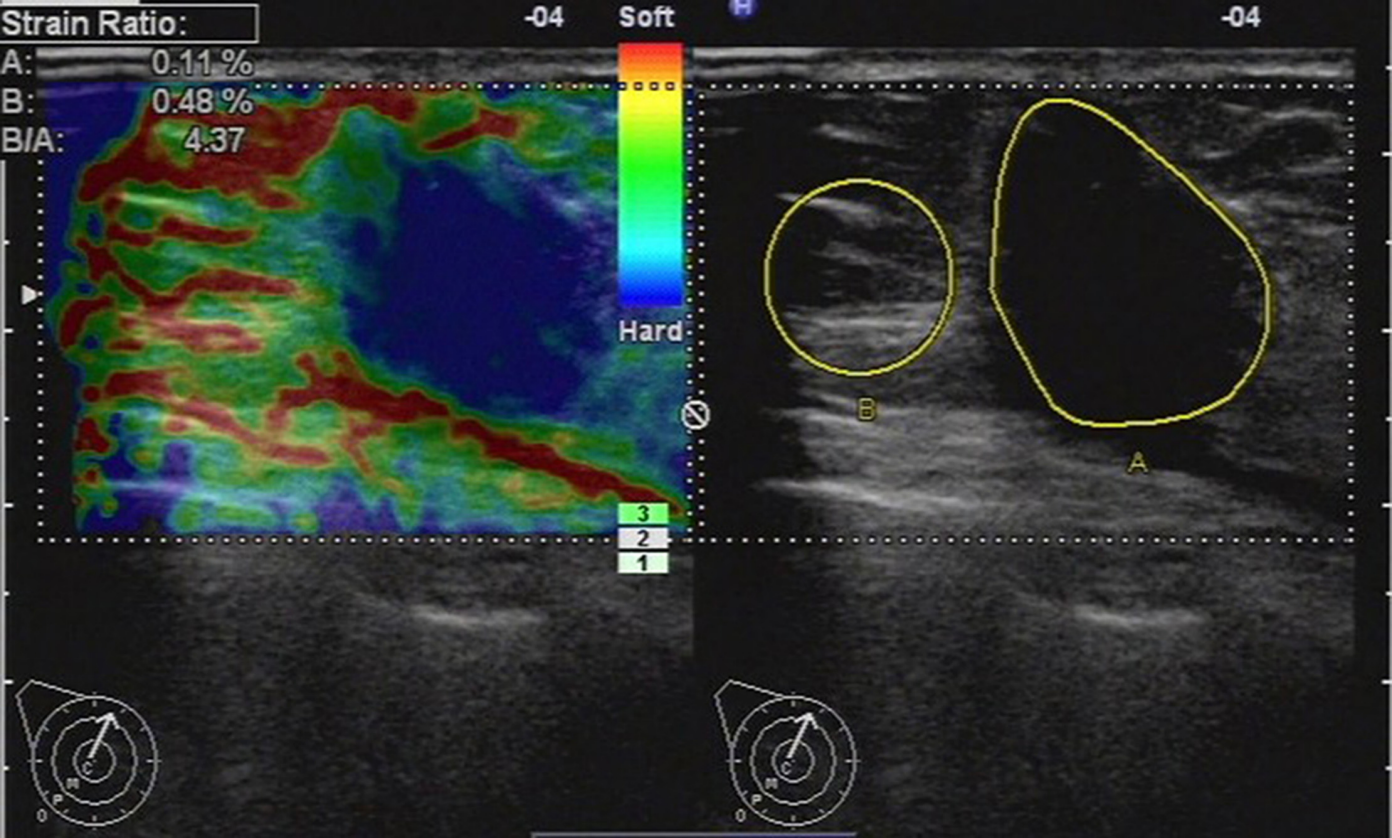
Ultrasonic elastographic image of a invasive lobular carcinoma in a 69-year-old woman. The lesion was scored 4 with the 5-point scoring method. The strain ratio was 4.37 with the strain ratio method at the same breast tissue depth as the reference.

486 lesions were included in the elasticity score 4 group. According to histology, 21.4% (104 of 486) were benign, among which 28 lesions were under strain ratio 3.01. 78.6% (382 of 486) were malignant, among which 337 lesions were exceeding strain ratio 3.01. Altogether the strain ratio method diagnosed 365 lesions (75.1%) correctly for score 4 group (Fig 9).

**Fig 10:**
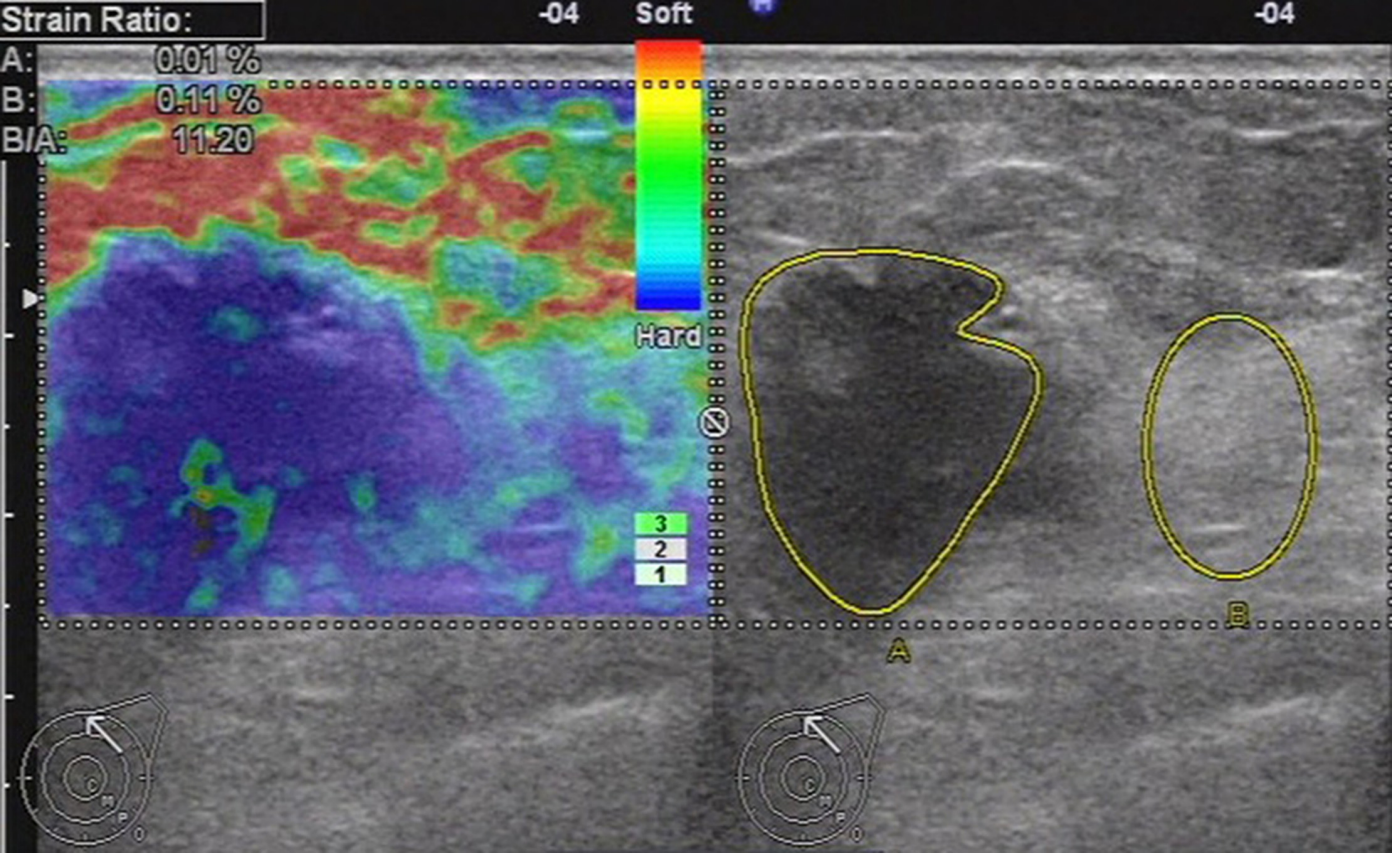
Ultrasonic elastographic image of a IDC in a 56-year-old woman. The lesion was scored 5 with the 5-point scoring method. The strain ratio was 11.20 with the strain ratio method at the same breast tissue depth as the reference.

48 lesions were included in the elasticity score 5 group. According to histology, 6.3% (3 of 48) were benign, among which no lesions were under strain ratio 3.01. 93.7% (45 of 48) were malignant, among which 44 lesions were exceeding strain ratio 3.01. Altogether the strain ratio method diagnosed 44 lesions (91.7%) correctly for score 5 group (Fig 10).

**Fig 11:**
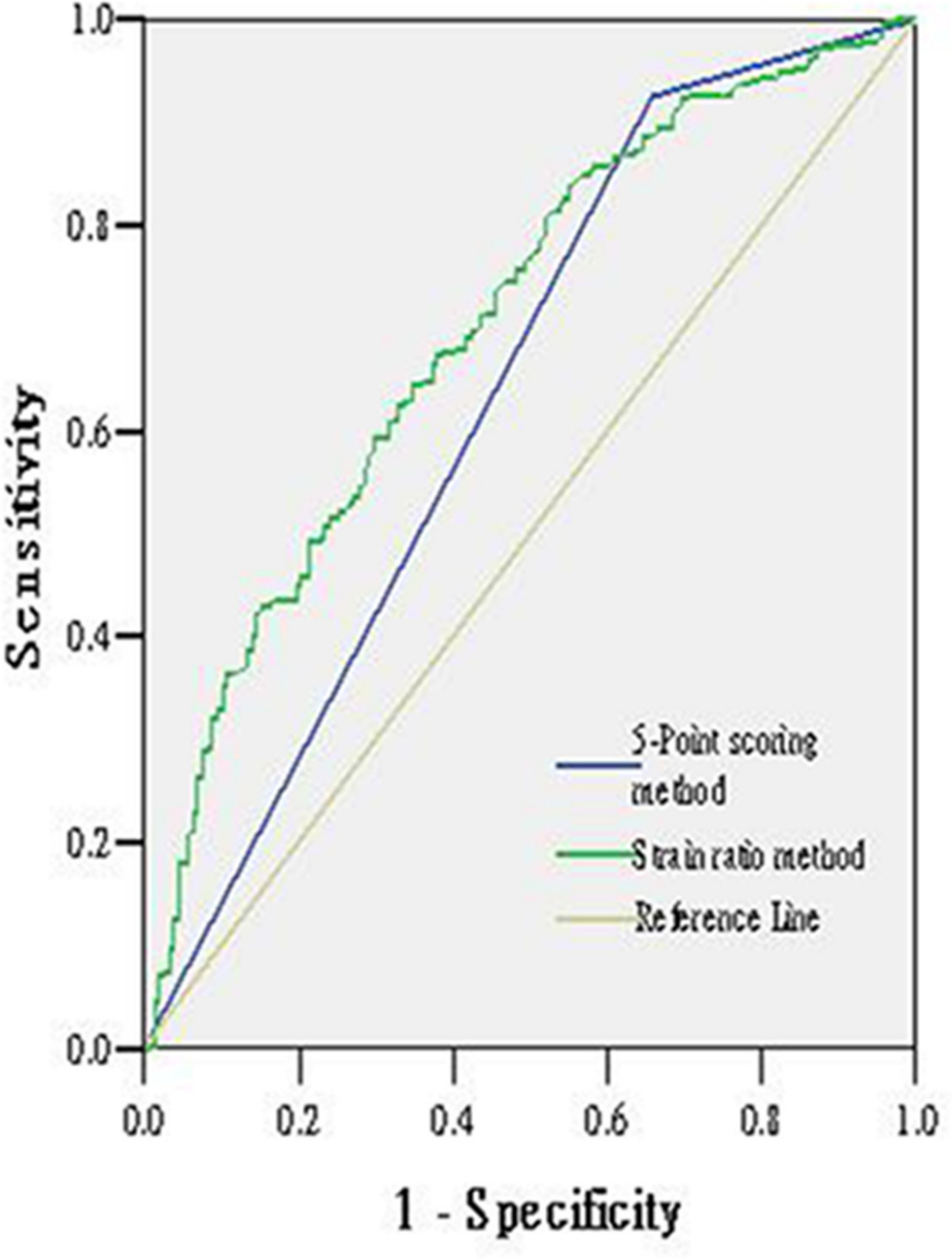
Receiver operating characteristic curves for the 5-point scoring method and the strain ratio method in differentiating malignant from benign legions for the lesions with elasticity score 3 and 4.

To take comprehensive assessment for the diagnosis performance of the two UE methods, the strain ratios were evaluated in conjunction with lesions of elasticity score 3 and score 4. For strain ratio method vs 5-scoring method, the performance results against histology were sensitivity 85.0% vs 92.4%(p = 0.02), specificity 43.0% vs 34.2%(p = 0.02), PPV 79.5% vs 78.6%(p = 0.46), NPV 52.3% vs 63.5%(p = 0.33), and accuracy 76.3% vs 73.4%(p = .002). According to the ROC curves (Fig 11), the AUC were 0.633 for 5-point scoring method and 0.703 for the strain ratio method (p = 0.01).

### False-negative and false-positive diagnosis with the strain ratio method

In this multi-center study, when the best cutoff point for the strain ratio was 3.01, the false-positive rate was 17.2% (100 0f 580) and the false-negative rate was 20.4% (102 of 500) by the strain ratio method. The histological types with false-negative and false-positive diagnosis by the strain ratio method are listed in Table 4. Furthermore, Table 5 shows the histological types of the lesions misdiagnosed by both the strain ratio method and the 5-scoring method simultaneously.

**Table 4:** Analysis of false-positive and false-negative diagnosis by strain ratio method.

False-positive (n = 101)	n	False-negative (n = 100)	n
Fibroadenoma	35	IDC	72
Papiloma	23	Ductal carcinoma in situ	18
Fibroadenomatous hyperplasia	18	Medullary carcinoma	3
Phyllodes tumor	12	Invasive papillocarcinoma	2
Chronic inflammation	4	Mucinous carcinoma	2
Atypical hyperplasia	2	Lymphoma	1
Others	7	Others	2

**Table 5:** Histological diagnosis with lesions misdianosed by both two UE methods.

Elasticity scores	Histological Diagnoses
1	IDC 1, Medullary carcinoma 1
2	IDC 2, DCIS 1
3	IDC 10, DCIS 2, Lymphoma 2
4	FA 12, Intraductal papiloma 8, Fibroadenomatous hyperplasia 4, Chronic inflammation 2, Atypical hyperplasia 2.
5	Intraductal papiloma 1, Phyllodes tumor 1, Fibrous tissue 1

## Discussion

As a noninvasive and cost-effective way for evaluating tissue stiffness of suspected breast nodules, the ultrasonic elastography performed successfully in differentiating between benign and malignant lesions. The 5-point scoring method is readily integrated into clinical practice since it suggested by Itoh et al[8]. But just as the B-mode sonography, this method contains observer-based variations during manipulation[13]. Therefore, Waki et al proposed that strain ratios could be as an evaluation system with more objective measurement methods which were in accordance with elasticity characteristic of pathological tissue. Yet the literature about adding strain ratio calculation or not is still controversy.[1,2,11,14,15]

With the cut-off point of 3.01, the stain ratio method perform successfully for differentiating between benign and malignant breast lesions. This is comparable to previous studies, which showed the specificity and sensitivity of of UE were 79–95.7% and 65.5–86.5%, respectively[7,8,16–18]. Our research found that the strain ratio method shows higher specificity (82.8%) than the 5-point scoring method (73.0%). However, the differences of other diagnostic index between the two UE methods were not statistically significant. As we also found similar diagnosis performance for differentiation of benign and malignant breast lesions between the two methods.

With regard to the comparison of the 5-scoring method and the strain ratio method, there are a lot of articles published before, our results are inconsistent with some of them, especially with most of researches at home[19,20], which got the result that the strain ratio method had a better diagnosis performance than the 5-point scoring method. In our study, we found similar diagnosis performance for differentiation of benign and malignant breast lesions between the two methods. Such inconsistency maybe because our study chosen the most suitable conditions to evaluate breast lesions of Chinese women. First, in this study, we use the new standard 5-point scoring method proposed by us before[13], based on a Chinese poly-center study, which may be more suitable for Chinese women. While in most other studies[19,20], the 5-point standard suggested by Itoh et al[8] was used. On account of the new evaluation system improving the diagnostic efficiency, the advantage of the strain ratio method is not relatively obvious. Second, in our study, we used breast glandular tissue at the same levels as the reference, when conducting strain ratios. That might contribute to obtain more accurate results. As Zhi et al[13] primarily used the breast glandular tissue at the same level as the reference point, we have the idea that superficial structures will be exposed to compression more than the deeper tissues in UE examination. Third, as a poly-center study implemented by 8 centers, we collected more than 1080 cases spread across the country, which presented a better database and got a comprehensive conclusion, especially for Chinese women.

In 2008, Lee JW et al reported that lesion size on US were predictor of invasion[21]. However, in 2012, Stachs A et al[22], revealed that the extent to which the diagnostic performance of strain ratio is dependent on the tumor size was unable to confirm. So in our study, further evaluation of diagnosis performance of strain ratios and elasticity scores methods in different size groups were done. We obtained consistent results with our previous study[19] that the strain ratio method had a better diagnostic performance than the 5-point scoring method in the detection of large solid breast lesions. This may be because the degeneration change in cells of the large lesion is more likely to happen than small ones, which means the tissue becomes increasingly inhomogeneous.

**Fig 12:**
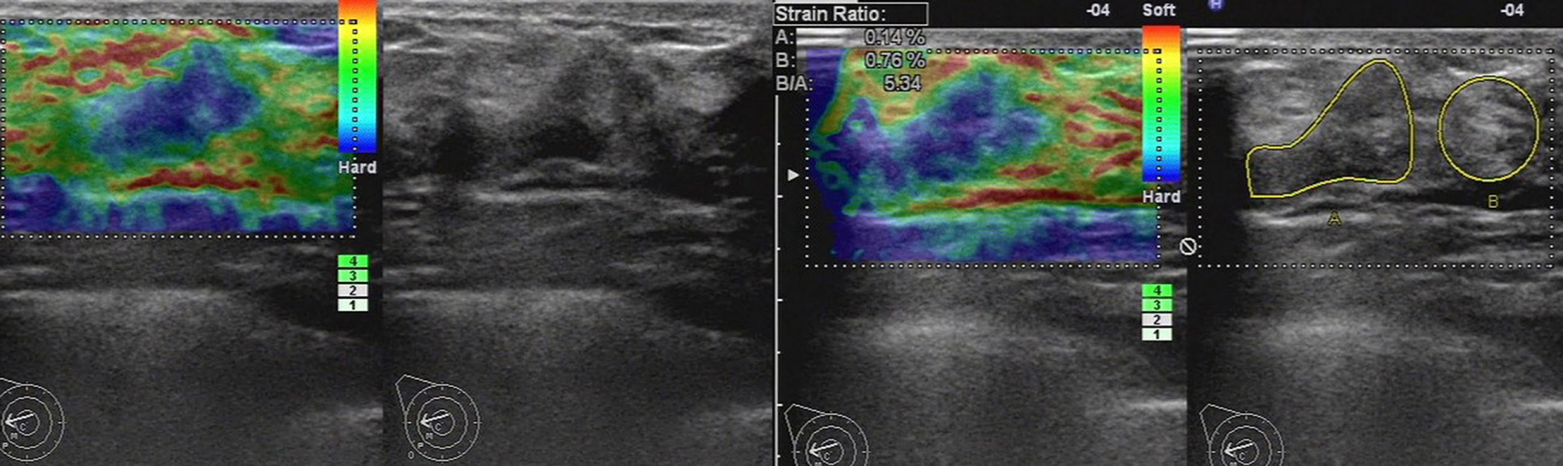
Ultrasonic elastographic images of a IDC in a 41-year-old woman. Left: The lesion was scored 3–4 with the 5-point scoring method. Right: The strain ratio was 5.34(malignant) with the strain ratio method at the same breast tissue depth as the reference.

When faced with the breast lesion, both the two UE diagnostic methods revealed a good correlation between strain ratios and elasticity score. The two UE evaluation systems showed consistency in the majority of breast lesions, especially for the lesions with elasticity score 1, 2 and 5. 98.0%, 88.8% and 93.0% of lesions with elasticity score 1, 2 and 5 got the same diagnosis with the strain ratio method respectively. While, 68.2% and 85.9% of lesions with elasticity score 3 and 4 got the same diagnosis with the strain ratio method. In clinical practices, the lesions with elasticity score 3 and 4 were difficult to determine than other scores, especially for the inexperienced observers. The reason was that when a color map was obtained, the decision of score 3 or 4 was just dependent on judging the ratio of blue and green (Fig 12), which in some extend asked for experience to give an accurate diagnosis. However, as a semi-parameter method, the strain ratios were calculated automatically by machine on the color map without observer interference. Therefore, the strain ratio method could contribute to differentiating benign and malignant breast lesions which were difficult to decide with the elasticity score. And as a more objective method, the inter-observer deviation would be avoided. In our study, there were 571 lesions with elasticity score 3 and 4. To take comprehensive assessment, we separately compared the two UE diagnostic methods for the lesions with elasticity score 3 and 4. And the strain ratio method shows better a diagnosis performance, which to our knowledge has not yet been reported.

In our study, we found that the strain ratios in the non-invasive breast cancer were lower than that in the invasive breast cancer, which may indicate the strain of neoplastic tissue is correlated with its malignant potential. That is similar to Nariya cho et al[23] who reported that elasticity score was the only independent predictor of invasion for the nonpalpable a DCIS.

**Fig 13:**
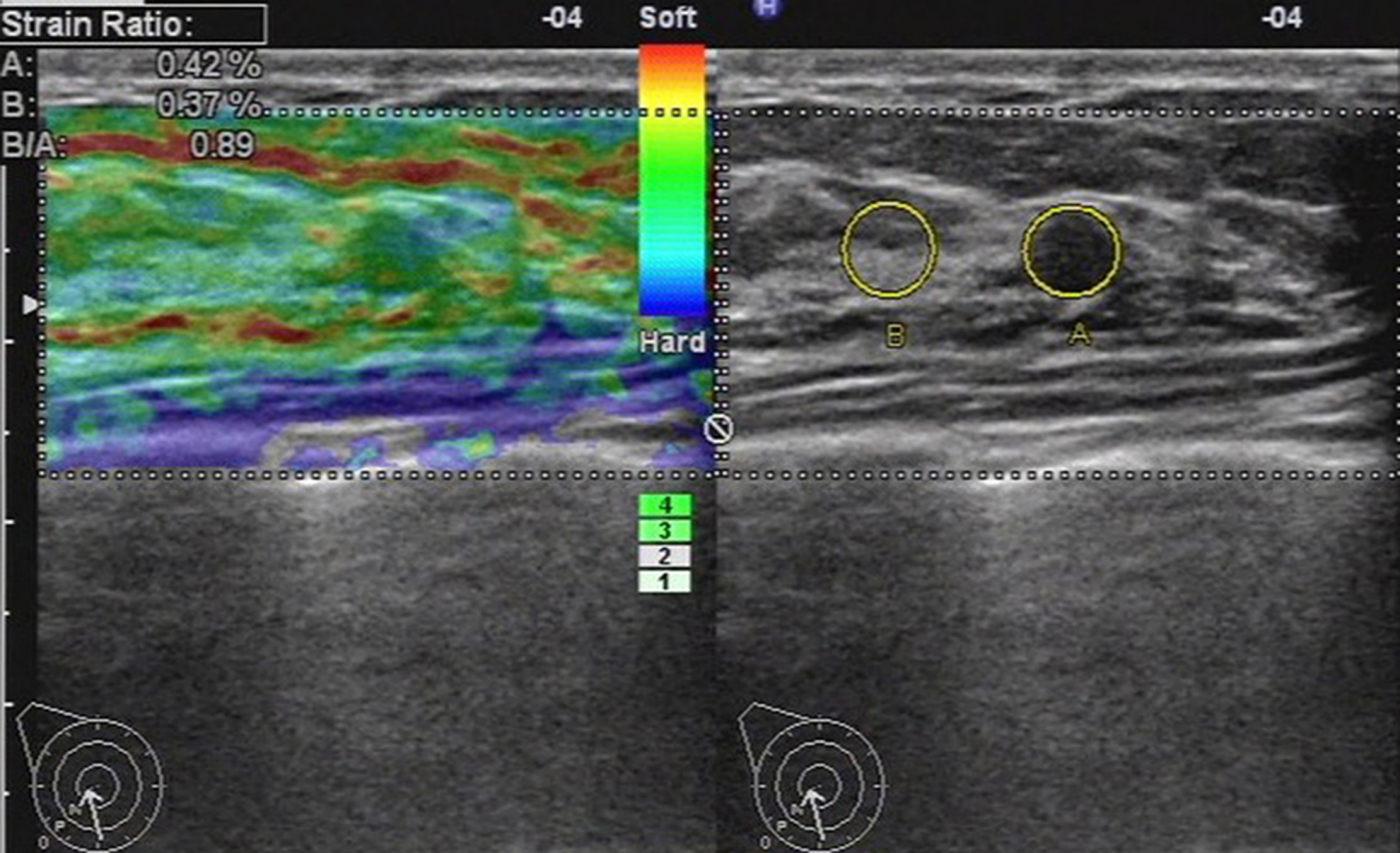
Ultrasonic elastographic image of a DCIS 32-year-old woman. The lesion was scored 1(benign) with the 5-point scoring method. The strain ratio was 0.89(benign) with the strain ratio method at the same breast tissue depth as the reference.

We also found that the strain ratio of malignant lesions was significantly higher than that of benign ones, but the overlap exited in elasticity coefficient for different tissues[24], so false-negative and false-positive results were unavoidable. The most of false-negative results of lesions misdiagnosis by both two UE methods simultaneously were invasive ductal carcinoma (IDC) and ductal carcinoma in situ (DCIS). In our study, majority of the malignant lesions were IDC. Although IDC was one of the most misdiagnosed histopathology, in fact, only 11.8% IDC were misdiagnosed. DCIS has been shown to be softer than IDC, which is consistent with other studies[23–25]. There were 53 DCIS lesions in our study and the mean strain ratio of them was 3.97±1.61, being between that of benign and malignant. 73.3% DCIS were misdiagnosed (Fig 13). Meanwhile, the most false-positive results misdiagnosis by both two UE methods simultaneously were intraductal papiloma. It is similar to Yi A et al[25], who suggested that the mean elasticity score of intraductal papiloma was higher than that of fibrocystic changes.

Our study had several limitations. First, the UE examine was taken after the B-mode examine in every case in our study. Namely, this evaluation style may cause an initial opinion based on B-mode sonography that might influence the UE diagnoses. Second, as a multiple center study, the inter-operator variability, when obtaining the color map by UE, is inevitable. Because the application of appropriate compression, which might vary by different examiners, could affect the color map[4,15]. Third, there is a little data lag in our research. Because as a poly-center study implemented by 7 hospitals, we concentrated the collected data which contained more than 1200 patient with more than 1400 lesions, screened and assessed every lesion was time-consuming.

## Conclusion

In our poly-center study, with the the best cutoff point 3.01, the stain ratio method performed successfully for differentiating between benign and malignant breast masses. And as a practical and objective technique, the strain ratio method could reduce subjective bias of observers. Although the 5-point scoring system and strain ratio system have similar diagnostic performance when faced with breast lesion, calculation of the strain ratios seems compulsory after evaluation of lesions with 5-point elasticity scoring, especially for the large solid breast lesions and the lesions with elasticity score 3 and 4.

## Supporting Information

S1 File The full institutional name of each separate IRB which evaluated and approved this study. (DOC)

## Abbreviations

UE: ultrasound elastography
BI-RADS: the Breast Imaging Recording and Data System
PPV: positive predictive value
NPV: negative predictive value
AUC: area under the ROC curve
IDC: invasive ductal carcinoma
DCIS: ductal carcinoma in situ
