# Supplementary material for: Ultrasonic Elastography Research Based on a Multicenter Study: Adding Strain Ratio after 5-Point Scoring Evaluation or Not
Source: PLoS One. 2016 Feb 10;11(2):e0148330. doi: 10.1371/journal.pone.0148330 (PMC4749284; doi:10.1371/journal.pone.0148330)
Supplement: S1 File — (DOC) [file pone.0148330.s001.doc]

**The full institutional name of each separate IRB which evaluated and approved this study:**

1.336 enrolled patients from Sun Yat-sen Memorial Hospital written and signed the informed consent which was approved by the Institutional Review Board of Ethic Committee of Zhongshan School of Medicine SYSU,Guangzhou, Guangdong Province, China.

2. 93 enrolled patients from West China Hospital of Sichuan University written and signed the informed consent which was approved by the Institutional Review Board of Ethic Committee of Sichuan University, Chengdu, Sichuan Province, China.

3. 281 enrolled patients from Huashan Hospital written and signed the informed consent which was approved by the Institutional Review Board of Ethic Committee of Fudan University, Shanghai, China.

4. 134 enrolled patients from Tumor Hospital Affiliated to Xinjiang Medical University written and signed the informed consent which was approved by the Institutional Review Board of Ethic Committee of Xinjiang Medical University,Urumqi, Xinjiang Province, China.

5. 94 enrolled patients from Xiangya Hospital written and signed the informed consent which was approved by the Institutional Review Board of Ethic Committee of Central-South University, Changsha, Hunan Province, China.

6. 68 enrolled patients from ShengJing hospitalwritten and signed the informed consent which was approved by the Institutional Review Board of Ethic Committee of China Medical University, Shenyang, Liaoning Province, China.

7. 31 enrolled patients from The First Affiliated Hospital of Harbin Medical University written and signed the informed consent which was approved by the Institutional Review Board of Ethic Committee of Harbin Medical University, Harbin, Heilongjiang Province, China.

8. 43 enrolled patients from Peking Union Medical College Hospital written and signed the informed consent which was approved by the Institutional Review Board of Ethic Committee of Chinese Academy of Medical Sciences and Peking Union Medical College, Beijing, Hebei Province, China.
